# Readdressing the Localization of Apolipoprotein E (APOE) in Mitochondria-Associated Endoplasmic Reticulum (ER) Membranes (MAMs): An Investigation of the Hepatic Protein–Protein Interactions of APOE with the Mitochondrial Proteins Lon Protease (LONP1), Mitochondrial Import Receptor Subunit TOM40 (TOMM40) and Voltage-Dependent Anion-Selective Channel 1 (VDAC1)

**DOI:** 10.3390/ijms251910597

**Published:** 2024-10-01

**Authors:** Johanna Rueter, Gerald Rimbach, Stephanie Bilke, Andreas Tholey, Patricia Huebbe

**Affiliations:** 1 Institute of Human Nutrition and Food Science, University of Kiel, Hermann-Rodewald-Strasse 6, 24118 Kiel, Germany; neuber@foodsci.uni-kiel.de (J.R.); rimbach@foodsci.uni-kiel.de (G.R.); 2 Institute of Experimental Medicine, University of Kiel, Niemannsweg 11, 24105 Kiel, Germany

**Keywords:** mitochondria–ER contacts, APOE4, stress response, chaperone, liver, co-immunoprecipitation, thapsigargin

## Abstract

As a component of circulating lipoproteins, APOE binds to cell surface receptors mediating lipoprotein metabolism and cholesterol transport. A growing body of evidence, including the identification of a broad variety of cellular proteins interacting with APOE, suggests additional independent functions. Investigating cellular localization and protein–protein interactions in cultured human hepatocytes, we aimed to contribute to the elucidation of hitherto unnoted cellular functions of APOE. We observed a strong accumulation of APOE in MAMs, equally evident for the two major isoforms APOE3 and APOE4. Using mass spectrometry proteome analyses, novel and previously noted APOE interactors were identified, including the mitochondrial proteins TOMM40, LONP1 and VDAC1. All three interactors were present in MAM fractions, which we think initially facilitates interactions with APOE. LONP1 is a protease with chaperone activity, which migrated to MAMs in response to ER stress, displaying a reinforced interaction with APOE. We therefore hypothesize that APOE may help in the unfolded protein response (UPR) by acting as a co-chaperone in cooperation with LONP1 at the interface of mitochondria and ER membranes. The interaction of APOE with the integral proteins TOMM40 and VDAC1 may point to the formation of bridging complexes connecting mitochondria with other organelles.

## 1. Introduction

The classical function of APOE is the solubilization and mediation of the binding of lipoproteins to cell surface receptors. Apart from that, there is an ever-growing wealth of evidence for additional roles of APOE, including its involvement in the stress response, immune function, the regulation of energy homeostasis, the response to dietary factors, and its nuclear role as a transcription factor (reviewed in [1,2]). The *APOE epsilon 4* (*APOEε4*) allele is a strong genetic risk factor of age-dependent Alzheimer’s dementia (AD) [3], which along with other forms of dementia affects over 50 million patients globally, costing the world economy over 14,000 billion international dollars [4]. Therefore, the majority of past APOE research concentrates on its role in neurodegeneration, and the elucidation of the molecular mechanisms underlying the *APOEε4*-disease risk association. The pursuit of causative factors and the connection to increased disease vulnerability has resulted in a biased perception that APOE4 is detrimental and inferior to APOE3, at least as far as brain metabolism is concerned. It must be considered, though, that the brain and the systemic circulation constitute separate APOE pools [5], with the main production site of peripheral APOE being the liver. Furthermore, opposed to its detrimental association with age-dependent morbidity and mortality [6,7], there is also evidence for advantages related to *APOEε4* including protection from fatty liver disease [8,9] or improved outcome in childhood diarrhea [10].

*APOEε4* is one of the three major allelic variants (*ε4*, *ε3* and *ε2*) that emerge from two nonsynonymous single nucleotide polymorphisms (SNP, rs429358 and rs7412), which render the APOE polymorphism unique to the human lineage. The SNP that distinguishes the disease-associated *ε4* from the most prevalent *ε3* allele causes an arginine-to-cysteine exchange and has been shown to impact denaturation stability and self-association properties (reviewed in [2,11]). Furthermore, the larger arginine and positively charged residue in the APOE4 protein alters the spatial conformation, increasing the exposure of the lipid binding region [12]. However, apart from occasional observations of delayed lipoprotein catabolism in the presence of APOE4 [13,14,15], receptor binding and lipid transport, the main functions of APOE seem not to be considerably different between APOE3 and APOE4.

Besides its presence along the secretory pathway, APOE has been assigned to other cellular compartments including the nucleus [16,17], the mitochondrion [18,19] and mitochondria–ER contact sites (MERCs) [20]. MERCs are maintained through the transient interaction between proteins of the outer mitochondrial membrane (OMM) and ER membranes, forming bridging complexes and creating a unique space for distinct biochemical processes and inter-organelle crosstalk [20,21]. For example, the OMM channel protein VDAC1 and the ER channel protein inositol 1,4,5-trisphosphate receptor type 1 (IP3R1) interact through the mediation of the chaperone glucose-related protein 75 (GRP75), enabling a rapid Ca^2+^ shuttle from the ER to the mitochondrion [22]. MERCs are highly dynamic and, amongst others, convey stress signals regulating autophagy, cell survival and apoptosis [23]. While MERCs may be regarded as punctiform conjunctions juxtaposing mitochondria and the ER at a fixed distance that is specific to the tethering protein complex, MAMs are the result of subcellular fractionation and are considered as a functional cellular compartment.

After their first description in 1990 [20], the distinct biochemical functions of MAMs and their participants faced slowly growing interest. By now, more than 1000 MAM-associated proteins have been identified by mass spectrometry proteomic analyses, exhibiting variable expression depending on the tissue examined [24]. APOE is part of the rank of MAM-associated proteins inferred from proteomic data [25,26], but the functional relevance of APOE in MAMs remains obscure. In this respect, the regulation of VLDL assembly [27] and phospholipid synthesis [28] have been proposed, as comprehensively reviewed recently [2]. New prospects on the elucidation of the biochemical function of MAM-associated APOE are provided by the observations that APOE not only interacts with the MAM-tethering protein VDAC1 [29,30], but that this interaction is also highly increased upon dietary restriction [31]. These data indicate that APOE accumulates in MAMs to participate in the regulation of cellular energy homeostasis and the stress response.

In addition to VDAC1, more than 150 APOE interactions have been archived in the BioGRID interaction database [32,33]. The comprehensive establishment of protein–protein interactions (PPIs) is crucial for a profound understanding of cellular mechanisms, and experimental validation complementing network-based computationally predicted interactions is mandatory in this process [34]. Although only a small number of APOE PPIs have been functionally examined to date, we have recently characterized the physical interaction between APOE and the mitochondrial branched-chain alpha-keto acid dehydrogenase subunit alpha (BCKDHA) in vivo. Using human APOE-targeted replacement mice, we investigated the differential impact of the two major isoforms APOE3 and APOE4 on the APOE-BCKDHA interaction, along with their interplay with dietary factors [31]. Building on our previous findings, we aim to further expand the knowledge surrounding APOE, and focus on cellular functions that go beyond the classic conception of APOE as a constituent of circulating lipoproteins. Readdressing its role in MAMs, we propose that APOE interacts with the OMM-localized proteins TOMM40 and VDAC1, potentially even as part of larger transport complexes, which may at least be indirectly related to MERC formation. The interaction with the mitochondrial chaperone and protease LONP1 appeared strengthened during the UPR, suggesting that APOE takes part in cellular proteostasis, potentially assisting in protein folding or degradation in a co-chaperone capacity.

## 2. Results

### 2.1. APOE Accumulates in the MAMs of Human Cultured Hepatocytes, Equally Evident for APOE3 and APOE4

**Figure 1:**
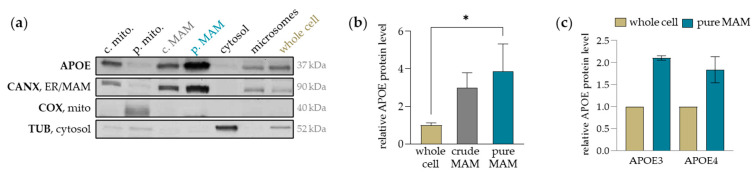
APOE accumulates in the MAMs of cultured hepatocytes, equally evident during APOE3 and APOE4 overexpression: (**a**) subcellular fractions were isolated from Huh7 cells by Percoll density gradient ultracentrifugation and analyzed by Western blotting. Representative images show the accumulation of the APOE protein in pure MAMs. Respective marker proteins were detected to visualize the purity of mitochondria (COX), MAMs (CANX) and cytosol (TUB); (**b**) APOE protein levels were quantified in the whole cell sample and crude and pure MAM fractions and normalized to the APOE level in the whole cell sample. In the pure MAM, the APOE protein level was significantly higher compared to the whole cell (*p* < 0.05, unpaired *t*-test) as indicated by an asterisk (*). The data are means ± SEM (*n* = 3); (**c**) no APOE isoform-dependent difference was observed in the accumulation of APOE in the pure MAM fraction in APOE3- and APOE4-transfected Huh7 cells. Data are means ± SEM (*n* = 2) and the accumulation of APOE in MAMs is related to the APOE protein level in the whole cell samples; subcellular fractions: c. mito., crude mitochondria; p. mito., pure mitochondria; c. MAM, crude mitochondria ER-membranes (MAMs), p. MAM, pure MAM.

To investigate the cellular localization of APOE, MAMs and other subcellular fractions were isolated from Huh7 cells. Figure 1a shows representative Western blot images of APOE and marker proteins for the corresponding cell organelles in the isolated subcellular fractions. The purity and enrichment of the individual fractions were visualized with corresponding marker proteins (CANX, ER membrane/MAM; COX, mitochondria; TUB, cytosol). APOE was significantly enriched fourfold in the pure MAM fraction, as compared to the whole cell protein level (Figure 1b). To determine whether APOE accumulation in MAMs is affected by the APOE isoform, MAM fractions were also isolated from APOE3- and APOE4-transfected Huh7 cells. APOE protein levels detected in the pure MAM fraction were normalized to APOE protein levels in the whole cell protein sample to quantify the subcellular accumulation. An approximate twofold APOE accumulation was observed in the pure MAM fraction of transfected cells, displaying no significant difference between the APOE3 and APOE4 isoform (Figure 1c).

Although a weak signal for APOE was observed in the pure mitochondrial fractions from unmodified Huh7 cells (Figure 1a), we believe this does not indicate mitochondrial localization but rather reflects incomplete separation from non-mitochondrial fractions. This is further supported by the detection of the cytosolic and ER membrane markers, tubulin and calnexin. In APOE-transfected cells, there were no clear mitochondrial bands seen in the Percoll gradient after the first ultracentrifugation step of the subcellular isolation procedure, as would be normally seen in unmodified cells. This indicates that the transient transfection reduces mitochondrial membrane integrity, hampering the separation of subcellular fractions, contributing to impurities and contaminations with non-specific proteins.

### 2.2. The Co-Localization of MERC-forming Proteins and Expression of MAM Markers Is Similar in the Presence of APOE3 and APOE4 in Cultured Hepatocytes and the Livers of Mice

**Figure 2:**
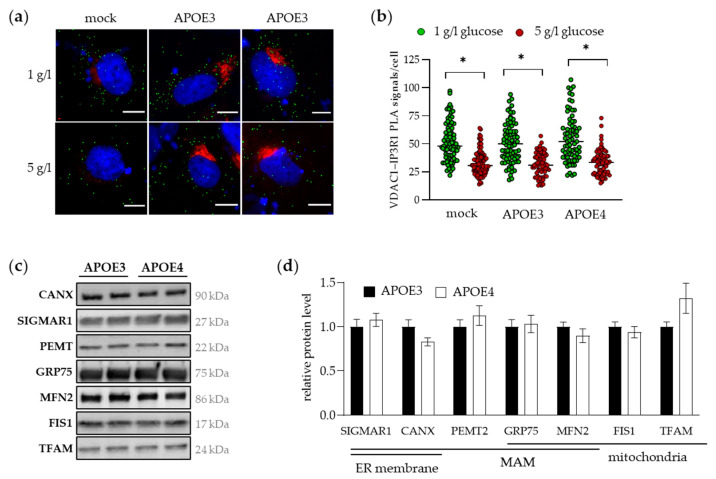
The number of visualized MERCs in cultured hepatocytes and MAM-assembling protein levels in the mouse liver are similar in presence of APOE3 and APOE4: (**a**) representative images of MERC-PLA experiments in APOE-transfected Huh7 cells that were incubated with 1 or 5 g/L glucose for six hours. VDAC1-IP3R1 PLA signals are shown in green, APOE was additionally stained in red to identify successfully transfected cells, and cell nuclei appeared blue by staining with DAPI. 400× magnification; scale bar 5 µm; (**b**) PLA signals from at least 100 cells per sample from three independent experiments were quantified showing a significant reduction of PLA signals per cell in all samples in response to the glucose challenge. No difference was observed comparing APOE3-, APOE4- and mock-transfected cells. Significance was accepted at *p* < 0.05, indicated with an asterisk (*); a two-way ANOVA was performed followed by the Šídák’s multiple comparisons test; (**c**) MAM-assembling as well as ER and mitochondrial marker proteins were analyzed in the livers of APOE-targeted replacement mice by Western blotting and representative images are shown; (**d**) densitometric analysis revealed no significant differences between APOE3 and APOE4 mice (unpaired *t*-test). Target band intensity was normalized by total protein load and related to the mean of APOE3 mice. Data are means ± SEM (*n* = 5–6).

Based on its prominent role within the MERC framework in facilitating the formation and stabilization of membrane contacts, the IP3R-GRP75-VDAC1 complex serves as a reliable marker for MAM integrity. To examine the effect of the APOE isoform on MERC formation, an in situ proximity ligation assay (PLA) was performed using antibodies against VDAC1 (OMM) and IP3R1 (ER membrane). Tubbs et al. [35] have demonstrated that the VDAC1-IP3R1 interaction can be regulated by the glucose concentration in the cell culture medium of Huh7 cells. Therefore, VDAC1-IP3R1 contacts were examined in APOE3- and APOE4-transfected Huh7 cells treated with 1 or 5 g/L glucose. Figure 2a shows representative images of the MERC-PLA experiment. PLA-positive signals are shown as green dots and APOE was additionally stained (in red) to distinguish successfully transfected from non-transfected cells. The nucleus was stained with DAPI. In all samples, incubation with 5 g/L glucose resulted in a significant reduction in contacts between the OMM (VDAC1) and the ER (IP3R1) compared to 1 g/L glucose (Figure 2b). No APOE isoform-dependent difference was observed as evidenced by similar numbers of MAM contacts per cell after both the 1 and the 5 g/L glucose treatment in APOE3- and APOE4-transfected cells. Overexpression of APOE in the APOE3- and APOE4-transfected Huh7 cells did not affect the number of mitochondria–ER contacts compared to the mock control (empty vector), which are cells expressing APOE endogenously.

To investigate whether APOE3 and APOE4 differentially affect MAM assembly in vivo, we resorted to the liver tissue of APOE3- and APOE4-targeted replacement mice. Because it is not possible to isolate pure subcellular fractions from frozen tissue samples, we examined the level of various mitochondrial and MAM proteins. Representative Western blot images and the corresponding densitometric analyses are shown in Figure 2c,d, demonstrating that there were no differences in protein levels of MAM-involved and mitochondrial proteins between APOE3- and APOE4-expressing mice.

### 2.3. Identification of TOMM40 and Other Mitochondrial and Non-Mitochondrial Proteins as APOE Interaction Partners in Human Cultured Hepatocytes

**Figure 3:**
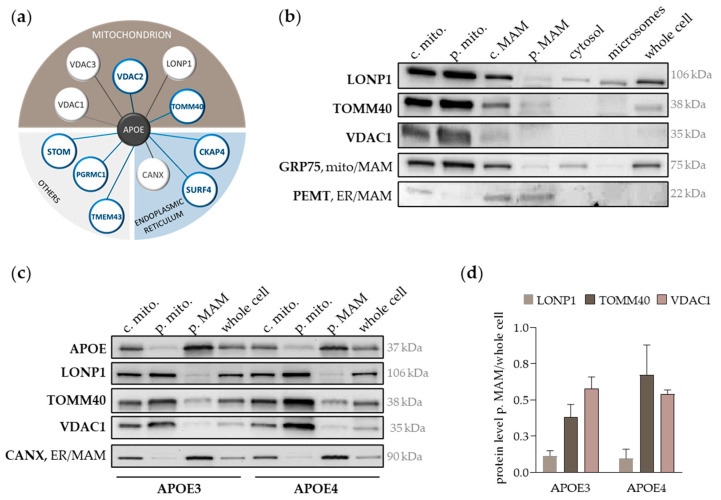
Mitochondrial APOE-interacting proteins are remotely connected to MAMs, with no apparent difference comparing APOE3- and APOE4-transfected cells. (**a**) Visualization of the eleven higher abundant protein–protein interactions (PPIs) shared by APOE3- and APOE4-transfected cells compared to unmodified Huh7 cells. PPIs were categorized by their main cellular localization identified according to The Human Gene Database GeneCards. Hitherto unknown PPIs (7/11) are highlighted by the greater thickness and blue color of the borderline. (**b**) Representative Western blot images of the presence of the APOE-interacting proteins LONP1, TOMM40 and VDAC1 in subcellular fractions isolated from Huh7 cells. Respective marker proteins for mitochondrial and ER-related MAM proteins were detected (GRP75, mitochondria, MAM; and PEMT, ER and MAM). (**c**) Representative Western blot images showing APOE, LONP1, TOMM40 and VDAC1 as well as the ER/MAM marker CANX in subcellular fractions of APOE3- and APOE4-transfected cells. (**d**) Target band intensities were quantified and normalized by total protein load per lane. The target protein level in the pure MAM fraction was related to the whole cell sample showing similar results in APOE3- and APOE4-transfected cells. Data are means ± SEM (*n* = 2). Subcellular fractions: c. mito., crude mitochondria; p. mito., pure mitochondria; c. MAM, crude mitochondria ER membranes (MAMs), p. MAM, pure MAM.

In a previous study, we identified 28 APOE-interacting proteins in the livers of targeted replacement mice expressing human APOE3 or APOE4 [31]. Distinct from this first approach, putative APOE protein interactions have now been identified within the proteome of human cultured hepatocytes (Huh7 cells). APOE-co-immunoprecipitation (co-IP) was performed either in unmodified Huh7 cells with endogenous APOE production (*APOE3/E3* genotype [36]) or in modified Huh7 cells transiently transfected with APOE3 and APOE4 overexpression vectors. Transfection with an empty vector (mock) served as a modification control and enables the evaluation of APOE protein interactions in response to cellular stress due to the transfection procedure. Promising PPI candidates were identified through the comparison of individual proteins lists with each other (Supplementary Table S1). We focused on the comparison with unmodified controls and determined twenty-one higher abundant PPIs in APOE3-transfected cells and thirteen in APOE4-transfected cells. Considering only those proteins significantly enriched in both comparisons and, thus, shared by APOE3 and APOE4, eleven APOE interactions were identified and are hereafter referred to as shared APOE interactions (Figure 3a). Four of them have been already annotated in the BioGRID interaction database [32,33] at this time, including the voltage-dependent anion-selective channel proteins 1 and 3 (VDAC1, VDAC3) and LONP1 [29,37]. Also, VDAC1, VDAC3 and LONP1 were among the identified APOE interactions in our preceding study in the mouse liver [31]. On the other hand, VDAC2 and TOMM40 were part of the seven newly identified APOE interactions (Figure 3a). According to The Human Gene Database GeneCards (only considering the highest confidence of localization), the mitochondrion was the most common cellular compartment (5 out of 11 shared APOE interactions), followed by the endoplasmic reticulum (ER) (3/11) (Figure 3a). While the high proportion of mitochondrial proteins among the identified APOE-binding partners appears remarkable, it has been noticed in previous studies, including ours [31]. For further experiments, we focused on three mitochondrial proteins, including LONP1, a soluble protease of the mitochondrial matrix, the novel candidate TOMM40, and VDAC1, most comprehensively described as a MAM protein and APOE interactor, among the candidates.

The interaction with LONP1, TOMM40 and VDAC1 was validated by Western blotting (Figure S1a) in independent co-IP samples from APOE3- and APOE4-transfected cells with normalization of the target band intensities to the corresponding APOE signal. Relative to APOE3-transfected cells, the APOE–protein interaction was slightly higher with TOMM40 (1.29-fold) and VDAC1 (1.20-fold) and, though with high variation, 2.11-fold higher with LONP1 in APOE4-transfected cells (Figure S1b). The higher extent of the interaction with mitochondrial proteins associated with APOE4 had no apparent effect on mitochondrial function, as measured by the ATP production rate in APOE3- and APOE4-expressing cultured hepatocytes (Figure S1c). Similarly, in the liver of targeted replacement mice, there was no difference between APOE3 and APOE4 mice in mitochondrial copy number (concentration of mtDNA) (Figure S1d), which is a biomarker of mitochondrial function in vivo [38].

VDACs and TOMM40 are both OMM residents, enabling the interaction with non-mitochondrial structures and proteins. As mentioned above, such a bridging complex formation has been well described for VDAC1 (IP3R-GRP75-VDAC1 [22]), but recently also for VDAC2 [39] and BAP31-TOMM40 [40], which connect ER membranes to mitochondria exerting specific MAM-inherent functions. In contrast, LONP1 is a soluble protein of the mitochondrial matrix. If the mitochondrial matrix was the sole cellular localization of LONP1, APOE would have to translocate to the mitochondria to enable the interaction of the two proteins. Aiming to elucidate a potentially shared cellular localization of APOE and its selected PPI candidates, we examined the levels of LONP1, TOMM40 and VDAC1 in subcellular fractions from cultured hepatocytes, in the same way as for APOE before.

### 2.4. LONP1, TOMM40 and VDAC1 Share Cellular Localizations with APOE, Particularly in MAMs, Which Was Equally Evident in APOE3- and APOE4-Transfected Cells

Unlike APOE, its interaction partners LONP1, TOMM40 and VDAC1 accumulate in pure mitochondria, while showing little to no presence in pure MAMs of unmodified Huh7 cells (Figure 3b). However, all proteins were abundantly present in the crude mitochondrial and MAM fractions, which also contain MAM proteins, as evidenced by the detection of PEMT and GRP75 (Figure 3b). LONP1 was additionally present in the cytosol and microsomes, with the latter serving as a further shared location with APOE apart from MAMs. Interestingly, the subcellular distribution of LONP1 resembled the pattern of GRP75, which is peripherally attached to MAMs, building a bridge between the two MERC tethering proteins VDAC1 (OMM) and IP3R (ER). Although LONP1 and GRP75 are both mitochondrially operating chaperones facilitating mitochondrial quality control through protein folding and degradation [41], LONP1 has also been linked to MAMs in the recent past [42].

Next, we investigated the cellular localization of our target proteins in response to the APOE isoforms in APOE3- and APOE4-transfected Huh7 cells. As observed for the APOE protein before, there was no marked APOE isoform-dependent difference in the subcellular distribution of LONP1 (Figure 3c). Calculating the accumulation of LONP1, TOMM40 and VDAC1 in pure MAMs (by normalizing to the signal in the whole cell sample), there was no difference between APOE3- and APOE4-transfected cells observable (Figure 3d). However, TOMM40 and VDAC1 seemed to be more present in pure and crude mitochondria of APOE4- compared to APOE3-transfected cells, which may explain the APOE isoform-dependent difference in PPI abundance (Table S1, APOE4 vs. APOE3_filtered). The more pronounced interaction of TOMM40 and VDAC1 with APOE in APOE4-transfected cells may simply result from the higher MAM-associated protein level and rather not be related to APOE4 isoform-specific alterations of spatial conformation or excess to potential binding sites.

Taken together, APOE, LONP1, TOMM40 and VDAC1 share a joint localization within or remotely associated to MAMs. To further investigate the role of MAMs in creating the necessary micro-environment for the selected APOE interactions, we determined the MAM-related presence and the extent of the PPI in cultured hepatocytes under varying cellular stress conditions, either strengthening or weakening mitochondria–ER contacts.

### 2.5. The UPR and ER Stress Provokes the Accumulation of APOE and LONP1 in MAMs, Strengthening Their Interaction

**Figure 4:**
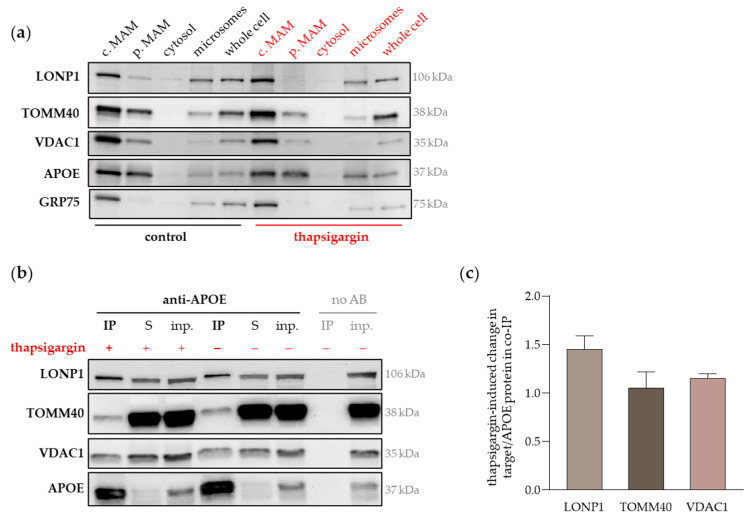
Presence in MAMs and extent of the PPI of selected candidates and APOE in ER-stressed cultured hepatocytes. (**a**) Western blot images of LONP1, TOMM40, VDAC1 and APOE detection in subcellular fractions of unmodified Huh7 cells. Thapsigargin treatment (50 µM, 24 h) provoked the accumulation of LONP1 and APOE in MAMs relative to the whole cell sample and compared to untreated control cells. GRP75 served as a marker for MAMs and the positive control for thapsigargin induced MAM protein translocation. c. MAM, crude mitochondria ER membranes (MAMs), p. MAM, pure MAM; (**b**) representative Western blot images of LONP1, TOMM40 and VADC1 in APOE co-IP samples from unmodified Huh7 cells treated with thapsigargin (50 µM, 24 h). No signals were visible in the IP negative control (no antibody used, no AB). S, IP supernatant, inp., input control; (**c**) target band intensity was normalized by the corresponding APOE band intensity. Relative target protein levels in thapsigargin-stressed cells were related to the mean of untreated cells (expressed as thapsigargin-induced change). Data are means from three individual cell culture experiments and co-IP (*n* = 3).

Thapsigargin is a pharmacological inhibitor of the ER protein folding machinery and leads to the accumulation of unfolded proteins and the stress response in the ER [43]. The UPR and ER stress are often followed by mitochondrial dysfunction, which has been related to the transmission of stress signals from the ER to mitochondria through an increasing number of mitochondria–ER contacts [44]. In order to preserve mitochondrial homeostasis, mitochondrial chaperones and other stress response proteins are recruited to MAMs to counteract the aggregation of unfolded proteins enhancing the formation of MERCs [42]. Subcellular fractions were isolated from unmodified Huh7 cells treated with thapsigargin (50 µM, 24 h) and protein levels were detected by Western blotting (Figure 4a, Supplementary Figure S2a). Target band intensity was normalized to total protein load per lane, followed by the calculation of target protein enrichment as relation to the corresponding whole cell sample. Thapsigargin treatment led to a 2.3-fold increase in the relative LONP1 accumulation in crude MAMs, only surpassed by the MAM marker GRP75, which displayed a 3.6-fold increase in crude MAM level, as compared to untreated control cells (Figure 4a). The effect of thapsigargin on crude MAM accumulation of TOMM40, VDAC1 and APOE was far less pronounced (1.05-, 0.77- and 1.02-fold change related to control, respectively). Since TOMM40 and APOE displayed strong signals in the pure MAM fraction, we also calculated thapsigargin-induced change in pure MAM accumulation. The accumulation of TOMM40 in pure MAM was clearly lessened (0.33-fold change) and of APOE marginally increased (1.23-fold change) comparing thapsigargin-treated with untreated cells.

Interestingly, the mitochondrial accumulation of LONP1, TOMM40, VDAC1 and GRP75 was apparently lower following ER stress induction (Supplementary Figure S2a), which may be a result of thapsigargin-triggered protein translocation or impaired mitochondrial integrity hampering the subcellular fractionation procedure and efficiency.

Next, we evaluated the extent of the PPPs during ER stress and determined the protein level of LONP1, TOMM40 and VDAC1 in APOE-co-IP samples from thapsigargin-treated Huh7 cells. The representative Western blot images in Figure 4b (experimental replicates in Supplementary Figure S2b) show that the LONP1/APOE protein level was increased in response to thapsigargin (1.5-fold), while the relative TOMM40/APOE and VDAC1/APOE levels were quite comparable to untreated control cells (Figure 4c). These data suggest that LONP1 and APOE at least partly interact within MAMs and thus, their interaction is dynamically responding to the modulation of MERC formation and cellular stress signals. Wondering whether the reduction of MERC formation would also impact on the APOE-LONP1 interaction, we challenged the unmodified Huh7 cells with glucose (5 g/L for 6 h) after a period of glucose shortage, in accordance with the experimental approach described above (Figure 2). Compared to cells kept under low glucose conditions (1g/L), there was a slight reduction (−20%) of the LONP1/APOE protein level (Supplementary Figure S2c), suggesting that the reduction of mitochondria–ER contacts also lowered the interaction of LONP1 and APOE. However, the APOE interaction with TOMM40 and VDAC1 appeared to be more or less unaffected by high glucose administration (1.01- and 1.12-fold change in target/APOE protein related to low glucose, respectively) and, thus, less sensitive to the reduction of MERCs as compared to LONP1 (Supplementary Figure S2c).

Taken together, the PPI of APOE with LONP1 was responsive to strengthening and weakening factors of MERC formation, while the interaction with TOMM40 and VDAC1 was relatively stable. Furthermore, LONP1 and APOE increased their accumulation in MAMs upon ER stress, which firstly may explain the higher extent of their interaction (assuming that MAMs are the major site of the PPI), and secondly suggests that their presence in MAMs, and possibly also their interaction, is part of the cellular stress response. The translocation of LONP1 from mitochondria to MAMs during the UPR has been recently emphasized [42,45], at least partly confirming our hypothesis.

## 3. Discussion

Based on our data and in line with earlier findings [20,25,26], APOE may reasonably be referred to as a MAM protein, although its resulting biochemical function remains to be elucidated. In addition to the lack of knowledge on the functional relevance of APOE in MAMs, any potential differences in response to the human APOE isoforms have not yet been systematically investigated. However, in the present study, there was no evidence pointing to differences in cellular localization and accumulation in MAMs of APOE, or in MAM assembly and function between APOE3 and APOE4, at least as far as liver cells are concerned. Previous studies in brain-derived cells, on the contrary, suggested that MAM activity and the number of MERCs are increased in the presence of APOE4 compared to APOE3 [28,46]. However, as the APOE isoform-dependent cellular localization and accumulation in MAMs have not been investigated in those studies, the observed enhancement of MERCs in response to APOE4 may also be due to other factors. Furthermore, as opposed to our analyses in hepatocytes and mouse livers, the APOE4-related modulation of MAM activity has been observed in neuronal cells and astrocytes [28,46]. The brain uses its own pool of APOE derived from glial cells, strictly separated from circulating APOE mainly derived from the liver [5,47]. The rigid compartmentalization of APOE pools likely includes the execution of tissue-specific functions [48] and beyond the tissue itself the cell type within the tissue also appears to be relevant for the functional comparison of APOE3 and APOE4 [49]. This may help to explain why, in the present study, hepatic MAM assembly was similar in presence of APOE3 and APOE4, whereas in brain-derived cells an apparent APOE4 isoform-dependent effect has been described [28,46].

Complementing our previous identification of putative APOE–protein interactions [31], we here determined both, yet unknown (e.g., TOMM40) and previously noted (e.g., LONP1, VDAC1) APOE-binding proteins, of which a great part possesses mitochondrial classification. APOE has been repeatedly linked to mitochondrial proteins and reported to translocate into mitochondria, although its major cellular localizations are the ER, nucleus and plasma membrane (reviewed in Rueter et al. [2]). Lacking a classical mitochondrion-targeting pre-sequence, the potential mitochondrial import mechanism of APOE is currently unknown. Therefore, the here newly identified interaction with TOMM40 is of special note, since the TOM complex is involved in at least three protein import pathways as alternatives to the classical targeting pre-sequence-mediated option [50]. One of these alternative pathways includes the binding of cytosolic and lysosomal proteins by chaperones of the heat shock protein (HSP) family followed by the mitochondrial import and further processing such as proteolytic cleavage and folding by mitochondrial proteases such as LONP1 [51,52]. The PPIs with TOMM40 and LONP1 may suggest that APOE translocates into mitochondria to be processed and properly folded, which would also imply that APOE exerts an essential mitochondrial function. In consequence, factors that affect the magnitude of the interaction with TOMM40 and LONP1 should impact on mitochondrial function, which may help to explain why APOE4 is related to mitochondrial impairment in brain-derived cells. However, the present study conducted in mouse livers and human hepatocytes lacked evidence of APOE isoform-dependent differences in mitochondrial function. The discrepancy between our observations in hepatocytes and those in the brain perfectly reflects the contrasting results concerning potential APOE isoform-dependent alterations in MERC formation, which has been discussed above. Nevertheless, aside from the lack of evidence for differences in hepatic mitochondrial function, subtle variations in protein binding were observed between APOE3 and APOE4. Proteome data suggested a stronger candidate interaction with APOE4; however, the Western blot analysis of independent co-IP replicates did not support this notion. The likely reason for the discordant results lies in the different approaches to normalize the data. Western blot results were expressed as prey-to-bait ratio, normalizing the intensity of LONP1, TOMM40 and VDAC1 to the respective APOE intensity detected in the IP sample. In contrast to this, proteome data were expressed as relative abundance of individual proteins comparing two different IP samples, neglecting potential differences in APOE expression or IP efficiency. Taking this into account, we chose not to overemphasize the observed trend of a stronger protein interaction with APOE4, and studied the impact of cellular stress on MAM localization and PPIs in cells expressing endogenous APOE. In view of our decision to set aside the APOE isoforms in the subsequent experiments, the systematic analysis of MAM-related activity comparing APOE2, APOE3 and APOE4 is required in the future.

Given the uncertainty surrounding the mitochondrial translocation of APOE, and the presence of its potential mitochondrial interactors in subcellular fractions containing MAMs, we propose that their interaction is more likely to occur outside the mitochondrion rather than within. Aiming to understand the inherent functional relevance, distinctive perspectives on the OMM channel proteins and the soluble protease LONP1 may prove helpful. The interaction between APOE and the transmembrane proteins VDAC1 and TOMM40 may be part of MAM tethering complexes, involving one or more other interactors. As a soluble protein, APOE could be involved in mediating the complex formation between OMM and ER membrane proteins, perhaps comparable to the role GRP75 plays in the IP3R-GRP75-VDAC1 complex. This hypothesis raises the question of which candidates among the identified APOE-interactors that reside in the ER membrane might be suitable. Calnexin (CANX) and the cytoskeleton-associated protein 4 (CKAP4) emerge as compelling targets, given their recent association with MAMs, particularly in the regulation of mitochondrial Ca^2+^ import [39,53]. Considering the distinct role of VDAC1 in MAM-related Ca^2+^ flux, exploring the potential complex formation between VDAC1 and either CANX or CKAP4, linked through APOE, could be a significant focus for future research. Furthermore, the interaction between APOE and mitochondrial proteins may likely be a part of large multimeric complexes, consisting of more than three components, and perhaps connecting mitochondria with more organelles than just the ER, for example with lysosomes. The next phases of research applying experimental techniques that overcome current limitations, e.g., the employment of cross-linking agents to preserve complex integrity, will decipher the multi-protein complexes associated with APOE in MAMs.

In contrast to the transport of small ions mediated by VDAC1, TOMM40 is part of the mitochondrial import channel system transporting nuclear-encoded proteins to the inner mitochondrial compartments. The binding of APOE to TOMM40 may point to a possible translocation pathway, but other scenarios are conceivable, including the possibility of an indirect association mediated by a third interactor. Extending this assumption, the observation that the amyloid precursor protein (APP) and its cleavage products bind to mitochondrial channel proteins, such as TOMM40 [54], is brought into new light. Proteolytic cleavage of APP and the following aggregation of amyloid-β (Aβ) peptides in the brain are major hallmarks of AD pathology [55]. Although the reason for the binding of APP and Aβ to mitochondrial channel proteins is unclear, it is implied to impair normal mitochondrial function and to contribute to neurodegeneration and disease pathology (reviewed by Reed et al. [56]). Meanwhile, APOE is regarded as a pathological chaperone, promoting the transport and clearance of Aβ peptides, and preventing amyloid aggregation [57,58]. When taken together, it may be considered that the interaction between APOE and TOMM40 results from indirect association through their independent binding to another protein or peptide, for example Aβ. Despite the fact that the APOE interactions could result from indirect binding, an essential purpose may still be served. For example, in view of its chaperone activity, APOE may bind and transport nuclear-encoded pre-processed proteins with mitochondrial localization. While its clients target the mitochondrial import machinery, APOE gets indirectly associated OMM proteins. Admittedly, in light of current evidence, this scenario remains largely conjectural. A thorough investigation of the functional relevance of APOE interactions in MAMs will be required in the future.

The interaction between APOE and LONP1 may be purely biophysical, but if an essential function is inherent, it may well be related to the UPR. To support this premise, LONP1 is a protease with chaperone activity, centrally involved in mitochondrial protein folding or degradation, acting in co-operation with GRP75 [41]. Therefore, LONP1 is upregulated in response to the accumulation of unfolded, oxidized and aggregated proteins in the mitochondria, as well as in response to impaired protein folding in the ER [59]. A growing body of evidence underlines the importance of extramitochondrial LONP1 localization, especially in MAMs, where LONP1 is involved in inter-organelle crosstalk, conveying stress signals, and orchestrates the cellular stress response [42,45,60]. In accordance, we noticed the distinct translocation of LONP1 to MAMs during ER stress response, which was not evident for TOMM40 and VDAC1. However, there was a modest accumulation in MAMs observable for APOE, potentially indicating a targeted recruitment comparable to LONP1 as part of the cellular stress response. As mentioned above, APOE is believed to engage chaperone activity through binding to aggregation-prone proteins and peptides, promoting their clearance [57,61]. It may reasonably be assumed that APOE collaborates with LONP1 in MAMs, assisting in the folding and degradation of accumulating unfolded proteins during ER stress. The stronger interaction between APOE and LONP1 in stressed cells may support this assumption, providing that the PPI is of functional relevance. Then, an involvement in sensing and transmission of stress signals that govern cell survival and death may also be conceivable. However, the increased magnitude of the interaction between APOE and LONP1 may, conversely, be incidental, resulting from extramitochondrial LONP1 translocation, elevating its accessibility to APOE, and further promoted by the accumulation of both proteins in MAMs. Once again, based on current evidence, we can only speculate on the functional relevance of the APOE-LONP1 interaction, as we cannot dismiss the possibility that it is purely biophysical and a secondary event of coincident localization. Finally, it may be considered that APOE associates with LONP1 as client rather than as co-operating chaperone, and is thus subjected to LONP1-mediated folding or proteolytic degradation. However, previous studies examining the aggresome in response to selective inhibition of LONP1’s chaperone activity [41,62] lacked evidence to substantiate the notion that APOE is targeted for LONP1-mediated folding, at least.

Taken together, MAMs are widely recognized as discretely operating functional entity, and with the present work, we wish to readdress the role of APOE as a MAM protein. We provide preliminary data indicating that APOE responds to ER stress through increased MAM translocation. The stronger interaction with LONP1 implies an enhanced collaboration under stressed conditions. Given the important role LONP1 plays in proteostasis, it is compelling to suppose that the interaction with APOE is of functional relevance in MAMs, involved in protein folding, degradation and inter-organelle crosstalk. Opposed to this, the interaction between APOE and the OMM proteins TOMM40 and VDAC1 remained relatively stable under varying cellular conditions. Assuming that APOE acts as chaperone for aggregation-prone proteins and peptides, it is possible that the association with OMM channel proteins results from indirect binding. In this context, the example of APP and Aβ has been presented, highlighting their interaction with mitochondrial proteins, including VDAC1 and TOMM40 [63]. It has been suggested that mitochondrial chaperones and proteases contribute to the general cellular proteostasis, including folding or proteolysis of proteins that are typically sparse in mitochondria [62]. Accordingly, it is supposed that APP and Aβ target mitochondria for their degradation and clearance [56], which possibly involves APOE for solubilization and transport, and leading to indirect binding of APOE to OMM import proteins. The exploration of composition of APOE-containing protein complexes and their potential functional relevance at the interface of mitochondria and other organelles will be the focal point of future research. Investigating the MAM proteome in the absence and presence of endogenous APOE, under varying cellular stress conditions, may serve as a valuable foundation. Advancing studies deciphering the composition of APOE-containing multimeric protein complexes, emphasizing the impact of isoform-dependent differences in protein conformation and binding affinity are warranted.

However, there is great significance to the selection of the experimental model system, which must be carefully considered, given the apparent discrepancy regarding APOE isoform-dependent cellular effects between varying tissues and cell types. For example, compared to APOE3, APOE4 has been associated with impaired mitochondrial function including lower ATP levels in the brain and brain-derived cells [64,65,66], which is not reflected by our present and previous [67] observations in the liver and cultured hepatocytes. A further key consideration is the role of the carcinogenic origin of many cultured cell lines, including Huh7 cells, which certainly impacts the outcome. Serving as a platform for autophagy and apoptotic pathways, MAMs regulate cell survival and death, and are implicated in carcinogenesis [68]. This is illustrated by the altered expression and localization of MAM proteins in cancer cells, with LONP1 being a notable example [69]. Therefore, a general conclusion concerning the role of MAM-located APOE and the significance of the interaction with LONP1, TOMM40 and VDAC1 should be drawn with due caution, due to the preliminary nature of our findings and experimental limitations of this study.

Future endeavors will expand upon our early insights to elucidate the role of APOE in MAMs that extends well beyond the traditional understanding as merely a component of secreted lipoproteins.

## 4. Materials and Methods

### 4.1. Cultivation of Human Hepatoma Cells

The human hepatoma cell line Huh7 (Institute of applied Cell Culture, Munich, Germany) was cultured in RPMI 1640 medium containing 2 mM L-glutamine, 1 mM sodium pyruvate, 4.5 g/L glucose, 10 mM HEPES, 1.5 g/L NaHCO3, 1% penicillin/streptomycin, and 10% fetal bovine serum (FBS; Gibco, Thermo Fisher Scientific, Waltham, MA, USA). Cells were grown in 5% CO_2_ at 37 °C under a humidified atmosphere. Except for the FBS, all cell culture reagents were purchased from PAN Biotech (Aidenbach, Germany). Glucose challenge was conducted by administration of 5 g/L glucose for 6 h after 24 h of low glucose (1 g/L) conditioned cell culture medium. Thapsigargin (Santa Cruz, Dallas, TX, USA) was applied at a final concentration of 50 µM for 24 h.

### 4.2. APOE3 and APOE4 Plasmid DNA Construction and Transformation and Transient Transfection of Human Hepatoma Cells

Plasmids containing human genes encoding APOE3 and APOE4 were used to study isoform-specific effects in cultured Huh7 cells. A detailed description of the construction of APOE3 and APOE4 plasmid DNA is given in Rueter et al. [31] and the transformation and plasmid DNA isolation were performed as described in Dose et al. [67]. An empty plasmid vector was used as a so-called mock control. The cells were seeded 24 h prior to transfection at 50–70% confluency. A ratio of 1.5–2 µL jetPEI DNA transfection reagent (VWR, Erlangen, Germany) per 1 µg plasmid DNA, diluted in 150 mM NaCl, was used to complex plasmid DNA. DNA-jetPEI-complexes were mixed, incubated at room temperature for 20 min, and added drop by drop to the cells. The cells were harvested 24 h after transfection for the PLA and seahorse assays. For the isolation of subcellular fractions and co-IP, the medium was changed after 24 h, and cells were harvested and processed 48 h after transfection.

### 4.3. Isolation of Subcellular Fractions

Subcellular fractions were isolated from Huh7 cells by differential centrifugation followed by Percoll density gradient centrifugation according to Williamson et al. [70], basic protocol 2, with slight modifications. Nearly confluent Huh7 cells (two 15 cm petri dishes per sample) were harvested by trypsinization, washed with Dulbecco’s phosphate-buffered saline (DPBS; PAN Biotech, Aidenbach, Germany), and resuspended in sucrose homogenization medium (250 mM sucrose, 10 mM HEPES; pH 7.4) containing protease inhibitors. Using a Dounce homogenizer with a tight glass pestle (D8938, Sigma, Steinheim, Germany), cells were homogenized carefully by shear forces and efficiency of homogenization was checked under the microscope. Homogenization was continued until >90% of the cells were broken and the homogenate was centrifuged at low g forces (600× *g*) to remove nuclei, cell debris and intact cells. An aliquot of post-nuclear supernatant was taken as a total protein control and stored at −80 °C for later Western blot analysis. Supernatants were stored overnight at 4 °C and centrifuged the next day at 10,300× *g*, 4 °C for 10 min, to pellet the crude mitochondrial fraction. The supernatant from this centrifugation was collected and ultracentrifuged at 100,000× *g* for 60 min at 4 °C in a Ti70 fixed-angle rotor (Optima L-90K Ultracentrifuge, Beckman Coulter, Krefeld, Germany) to purify microsomes (pellet) as well as cytosolic fractions (supernatant). Crude mitochondria pellets were washed, resuspended in mannitol buffer A (250 mM mannitol, 0.5 mM EGTA, 5 mM HEPES; pH 7.4), layered drop by drop on 30% isotonic Percoll in mannitol buffer B (225 mM mannitol, 1 mM EGTA, 25 mM HEPES; pH 7.4), and ultracentrifuged at 95,000× *g* for 65 min at 4 °C in a SW40 swinging bucket rotor (Optima L-90K Ultracentrifuge, Beckman Coulter, Krefeld, Germany). Deceleration and acceleration profiles were set on “slow”. After ultracentrifugation, the MAM and mitochondrial fractions were carefully collected from the self-generated Percoll density gradient. To remove remaining Percoll from collected fractions, samples were diluted in ice-cold DPBS and centrifuged at 6300× *g* at 4 °C. The pellet of the mitochondrial fraction (pure mitochondria) was captured and transferred into a new cup. The supernatant of the MAM fraction was transferred to a new ultracentrifuge tube and ultracentrifuged at 100,000× *g* for 30 min at 4 °C in the fixed angle rotor. Approximately 200 µL of a loose pellet of pure MAM was captured from the bottom of the ultracentrifugation tube and transferred into a new cup. The MAM pellet from the 6300× *g* centrifugation step was resuspended in DPBS; this fraction contains crude MAM. All fractions were supplemented with protease inhibitors and stored at −80 °C until further analysis. The protein content of isolated fractions was measured with the Pierce BCA Protein Assay Kit (Thermo Fisher Scientific, Waltham, MA, USA) and equal amounts of protein were loaded on polyacrylamide gels for Western blotting. The isolated fractions were validated for enrichment and clear distinction among them, detecting organelle specific marker proteins with distinguishable banding patterns [71]: COX, as exclusively mitochondrial protein; CANX and PEMT, as ER membrane markers and known integral MAM proteins; and GRP75, which is a soluble mitochondrial protein described as peripherally associated to MAMs.

### 4.4. Co-Immunoprecipitation (co-IP)

Cells were lysed in either NP40 lysis buffer (150 mM sodium chloride, 50 mM TRIS, 5 mM EDTA, 1% NP40) or RIPA buffer (150 mM sodium chloride, 50 mM TRIS, 1% NP40, 0.5% sodium deoxycholate, 0.1% SDS) containing protease inhibitors depending on the subsequent analysis (proteome analysis or Western blotting). The protein concentration was measured with the Pierce BCA Protein Assay Kit (Thermo Fisher Scientific, Waltham, MA, USA) ensuring that equal amounts of protein (ranging between 770–1000 µg) were used for the co-IP. Cell lysates were initially precleared with Protein G Sepharose beads (GE Healthcare, Uppsala, Sweden) and then subjected to APOE precipitation using 5 µg of the anti-APOE antibody (sc-13521, Santa Cruz, Dallas, TX, USA) overnight at 4 °C. Negative controls using either no antibody (no AB) or an antibody isotype control (anti-mouse IgG1, 66360-1-Ig, Proteintech, Planegg-Martinsried, Germany) were also established. Input controls were taken from the pre-cleared samples (containing 2% of the protein amount) prior to the IP. The full procedure can be extracted from Rueter et al. [31].

### 4.5. Proteome Analysis

To identify APOE PPIs in human cultured hepatocytes, proteomic quantification was performed with four individual co-IP samples obtained from either (i) unmodified Huh7 cells, or Huh7 cells transfected with an: (ii) APOE3-overexpressing vector, (iii) APOE4-overexpressing vector, or (iv) empty vector = mock. The full procedure including sample preparation, MS conditions and raw data processing can be extracted from the Supplementary Information. The relative enrichment of APOE-interacting proteins was calculated comparing the lists of identified peptides of the four IP samples with each other highlighting significant candidates that were at least twofold more abundant.

### 4.6. APOE-Targeted Replacement Mice

APOE-targeted replacement mice expressing human APOE isoforms served as model to examine the effect of APOE3 and APOE4 in vivo. The mouse model and the protocol for the study are described in Rueter et al. [31]. Briefly, male APOE3- and APOE4-targeted replacement mice (Taconic Europe A/S, Ry, Denmark) (six per group) were held under constant environmental conditions (12 h light/dark cycle, 22 °C, 55–60% humidity) and had free access to water and food. The mice were fed a semisynthetic diet rich in fat and sugar (TD88137 modified, Ssniff, Soest, Germany). One APOE4 mouse dropped out and had to be euthanized in compliance with animal welfare regulations. At 9–10 months of age, the mice were killed and their livers were removed, snap-frozen in liquid nitrogen, and stored at −80 °C until analysis.

### 4.7. Western Blotting

Protein expression in whole-cell liver lysates (described in Rueter et al. [31]) from APOE3- and APOE4-targeted replacement mice, co-IP samples of and subcellular fractions isolated from Huh7 cells were analyzed by Western blotting as previously described [67]. The target proteins were detected using the primary antibodies listed in Table 1.

### 4.8. Proximity Ligation Assay (PLA)

The PLA was conducted in situ in intact fixed Huh7 cells to detect contact sites between mitochondria and the ER membrane [72]. Cells were seeded in 24-well plates on glass cover slips coated for 20 min with 0.01% poly-L-lysine (Sigma, Steinheim, Germany) and transfected in RPMI 1640 medium containing 1 g/L glucose 24 h after seeding. Twenty-four hours after transfection and prior to the PLA assay, the cells were incubated for six hours with low (1 g/L) and high (5 g/L) glucose concentrations, respectively. Both, the low and high glucose media were prepared by adding glucose to glucose-free RPMI 1640 medium (Gibco, Thermo Fisher Scientific, Waltham, MA, USA). After the glucose treatment, fixation of the cells was carried out using 4% paraformaldehyde in DPBS, incubated for 15 min at room temperature. Cells were washed three times with DPBS and permeabilized with 0.1% NP40, 100 mM glycine in DPBS for 30 min at room temperature. The PLA was performed according to the manufacturer’s instructions using the Duolink in situ detection reagents green, wash buffers, and appropriate combinations of mouse and rabbit PLA probes. All Duolink and PLA reagents were purchased from Sigma (Steinheim, Germany). The cells were blocked with the Duolink blocking solution overnight at 4 °C, followed by incubation for 60 min with mouse anti-inositol 1,4,5-trisphosphate receptor type 1 (IP3R1) (sc-271197, Santa Cruz, Dallas, TX, USA) and rabbit anti-VDAC1 (55259-1-AP, Proteintech, Planegg-Martinsried, Germany) primary antibodies, both diluted 1:100 in Duolink antibody diluent, at room temperature. PLA probes corresponding to the species of primary antibodies were diluted in Duolink antibody diluent, applied to the cover slips, and incubated for 60 min at 37 °C. Then, the cells were incubated with ligase (30 min, 37 °C), polymerase (100 min, 37 °C) and fluorescent anti-APOE antibody (1:20, 90 min, room temperature; sc-13521 AF546, Santa Cruz, Dallas, TX, USA). The cover slips were repeatedly washed with Duolink wash buffer A or B between the individual incubation steps and protected from light since the reagents are light-sensitive. In the last step, the cover slips were mounted with Duolink in situ mounting medium with DAPI onto slides and sealed with nail polish after 15 min. PLA signals were detected and images acquired using a Zeiss Axio Observer D1 Inverted Fluorescence Microscope and AxioVision software version 4.8 (Carl Zeiss Microscopy, Oberkochen, Germany). The images were analyzed and edited with ImageJ 1.8.0 [73] and, for the analysis, the PLA images were converted into binary black and white images and the particles per cell were counted and expressed as PLA signals per nuclei. After transfection, the endogenous APOE genotype of the Huh7 cell line (APOEε3/ε3, according to [36]) was overwritten by overexpressed APOE, which rendered actually homogeneous APOE3- or APOE4-transfected Huh7 cells. By additional staining of APOE, distinguishing cells overexpressing APOE from those with endogenous APOE expression was enabled. Thus, only successfully transfected cells were considered and investigating the potential APOE isoform-dependent effect was enabled.

### 4.9. Statistical Analysis

GraphPad Prism 9.1.0 (GraphPad Software, La Jolla, CA, USA) was used for statistical calculations. Data were tested for normality of distribution using the Shapiro‒Wilk test and for homogeneity of the variances using the Brown‒Forsythe test. For comparison of two groups, an unpaired *t*-test (parametric data) or Mann‒Whitney U test (non-parametric data) was conducted. A two-way ANOVA followed by Šídák’s post hoc multiple comparison was performed to analyze the data from the PLA experiment and Seahorse assay. All results are shown as means ± SEM. Significance was accepted at *p* < 0.05 and indicated with an asterisk (*) in the figures.

## Figures and Tables

**Table 1:** Antibodies used for Western blot protein detection.

Protein		Antibody	Company
APOE	Apolipoprotein E	sc-13521	Santa Cruz, Dallas, TX, USA
CANX	Calnexin	ab13504	Abcam, Cambridge, UK
COX	Cytochrome C oxidase	ab110413	Abcam, Cambridge, UK
GRP75	Glucose-regulated protein 75	ORB214063	Biorbyt, Cambridge, UK
FIS1	Mitochondrial fission 1 protein	10956-1-AP	Proteintech, Planegg-Martinsried, Germany
LONP1	Lon protease	15440-1-AP	Proteintech, Planegg-Martinsried, Germany
MFN2	Mitofusin 2	12186-1-AP	Proteintech, Planegg-Martinsried, Germany
PEMT	Phosphatidylethanolamine N-methyltransferase	ORB46023	Biorbyt, Cambridge, UK
SIGMAR1	Sigma non-opioid intracellular receptor 1	15168-1-AP	Proteintech, Planegg-Martinsried, Germany
TFAM	Mitochondrial transcription factor A	sc-166965	Santa Cruz, Dallas, TX, USA
TOMM40	Translocase of outer mitochondria membrane 40	18409-1-AP	Proteintech, Planegg-Martinsried, Germany
TUB	Tubulin	ab7291	Abcam, Cambridge, UK
VDAC1	Voltage-dependent anion-selective channel 1	sc-8828	Santa Cruz, Dallas, TX, USA

## Data Availability

The mass spectrometry proteomics data have been deposited to the ProteomeXchange Consortium via the PRIDE [76] partner repository with the dataset identifier PXD055281.

## Supplementary Materials

The following supporting information can be downloaded at: https://www.mdpi.com/article/10.3390/ijms251910597/s1. Refs. [74,75] are cited in Supplementary Materials file.
