# Supplementary material for: Readdressing the Localization of Apolipoprotein E (APOE) in Mitochondria-Associated Endoplasmic Reticulum (ER) Membranes (MAMs): An Investigation of the Hepatic Protein–Protein Interactions of APOE with the Mitochondrial Proteins Lon Protease (LONP1), Mitochondrial Import Receptor Subunit TOM40 (TOMM40) and Voltage-Dependent Anion-Selective Channel 1 (VDAC1)
Source: Int J Mol Sci. 2024 Oct 1;25(19):10597. doi: 10.3390/ijms251910597 (PMC11476584; doi:10.3390/ijms251910597)
Supplement: Supplementary file 1 [file ijms-25-10597-s001.zip › ijms-3209768-supplementary.pdf]

Supplementary information

Readdressing the Localization of Apolipoprotein E (APOE) in Mitochondria-Associated Endoplasmic Reticulum (ER) Membranes (MAMs): An Investigation of the Hepatic Protein–Protein Interactions of APOE with the Mitochondrial Proteins Lon Protease (LONP1), Mitochondrial Import Receptor Subunit TOM40 (TOMM40) and Voltage-Dependent Anion-Selective Channel 1 (VDAC1)

Johanna Rueter, Gerald Rimbach, Stephanie Bilke, Andreas Tholey and Patricia Huebbe

Results

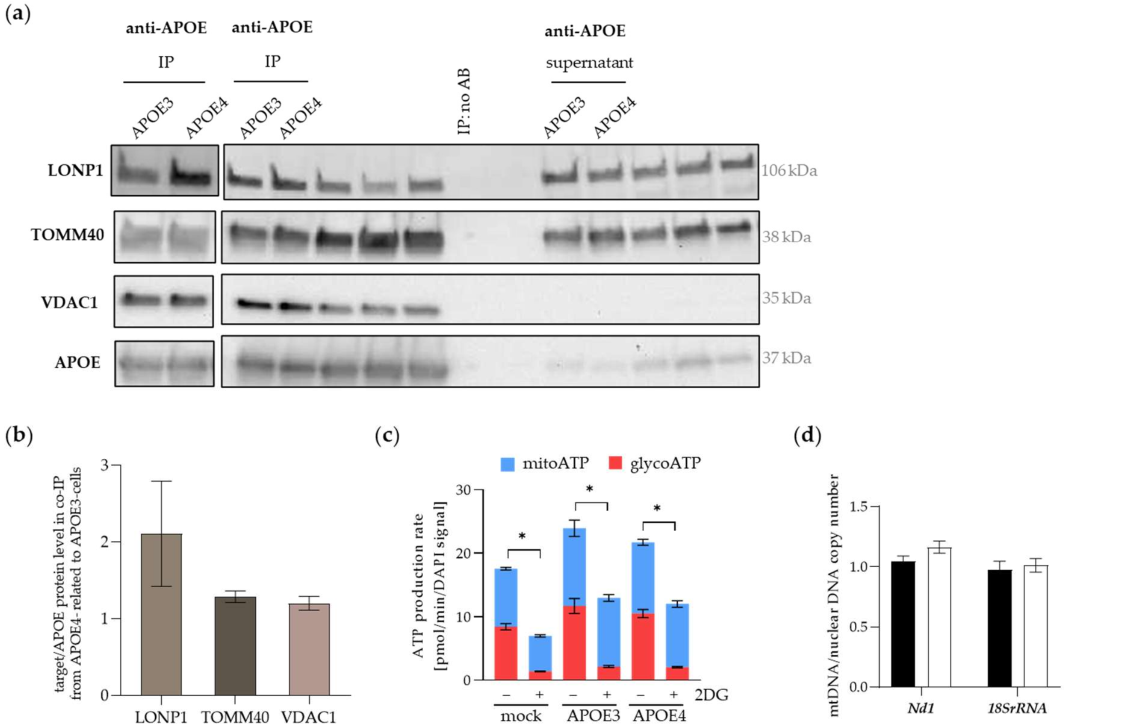

Figure S1. APOE isoform-dependent interaction with mitochondrial target proteins does not affect biomarkers of mitochondrial function. (**a**) Western blot images of LONP1, TOMM40, VDAC1 and APOE detection in APOE co-IP samples from APOE3- or APOE4-transfected Huh7 cells. No target bands were visible in the negative control (no AB).; (**b**) Target band intensities were normalized to the corresponding APOE band. Relative protein levels in APOE4-transfected cells were related to the mean values from APOE3-transfected cells. Data are means ± SEM (n=2). (**c**) The real-time ATP production rate in APOE-transfected cells was analyzed using the Seahorse technology with subsequent cell count normalization as measured by fluorescence of DAPI-stained cell nuclei. Glycolysis-derived ATP (glycoATP) was overall decreased by 2-deoxyglucose (2DG) treatment. Relative levels of ATP from mitochondria (mitoATP) and glycoATP were not different between APOE3- and APOE4-transfected cells. Data are means ± SEM (n=5-6). For statistical analysis, a two-way ANOVA was performed followed by the Sídák's multiple comparisons test and significant differences ($p<0.05$) were indicated with an asterisk (*). (**d**) mitochondrial DNA (mtDNA) concentration was determined in livers of APOE-targeted replacement mice by qPCR. Copy numbers of

the mitochondrially encoded genes *Nd1* and *18SrRNA* were normalized to the nuclear encoded housekeeping genes *Hk2* and *Gapdh*. Hepatic mtDNA concentration was not different between APOE3- and APOE4-targeted replacement mice. Data are means ± SEM (n=5-6). A t test for independent samples was performed accepting significance at $p<0.05$.

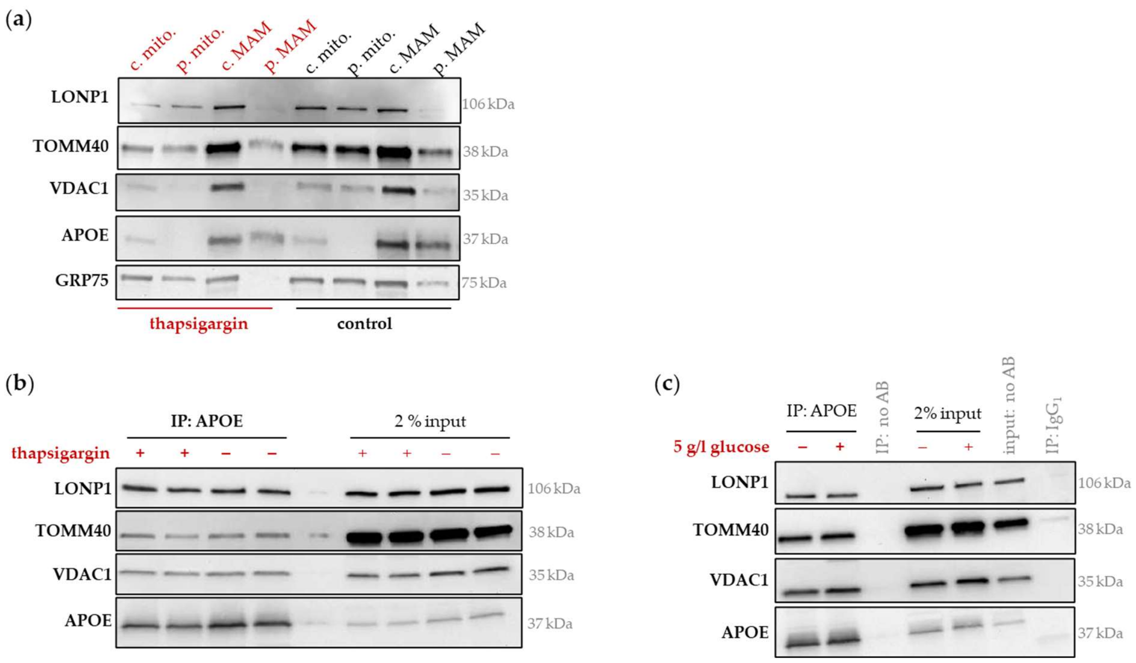

Figure S2. Detection of LONP1, TOMM40 and VDAC1 in mitochondrial and MAM fractions and APOE co-IP samples of unmodified Huh7 cells in response to different stress conditions. (**a**) Western blot images of target protein detection in mitochondrial and MAM fractions originating from the same fractionation procedure yielding the fractions used for independent Western blotting depicted in Figure 4a (main text). Pure mitochondrial protein levels were lower in thapsigargin-treated cells (50 μM, 24 h) compared to untreated control. Crude (c.) and pure (p.) mitochondrial (mito.), mitochondria-associated ER membranes (MAM); (**b**) Western blot images of LONP1, TOMM40, VDAC1 and APOE in APOE co-IP samples from thapsigargin-treated cells (50 μM, 24 h). These are experimental replicates corresponding to the data depicted in Figure 4b (main text). (**c**) Western blot images of LONP1, TOMM40 and VDAC1 in APOE co-IP samples of unmodified Huh7 cells challenged with glucose. A marginal reduction of LONP1 was observable in comparison to unchallenged cells kept under low glucose conditions (1 g/l). Negative IP controls were performed in the absence of the APOE antibody (no AB) and using an isotype control (IgG_1) showing no or only faint signals.

Materials and Methods

Proteome analysis

Co-IP samples were purified by chloroform-methanol-water precipitation. Precipitates were resuspended in 100 mM ammonium bicarbonate (ABC), reduced with 100 mM dithiothreitol (56 °C, 1h), alkylated with 400 mM iodoacetamide (in the dark, rt, 30 min) and digested with 1:50 (200 ng/μL) trypsin (37 °C, overnight). The samples were cleaned and desalted with C18-ZipTips, dried in a vacuum centrifuge and resuspended in 15 μl 0.1 % trifluoroacetic acid (TFA) in 3 % acetonitrile (ACN).

Samples were analysed on an UltiMate 3000-RSLCnano system (Thermo Fisher Scientific, Waltham, MA, USA) which was equipped with an Acclaim PepMap 100 C18 column (3 μm, 100 Å, 150 mm × 75 μm) and coupled to a QExactive Plus MS system. Eluent A (0.05 % formic acid (FA)) and eluent B (0.05 % FA, 80 % ACN) were used for separation at a flow rate of 300 nL/min and a 90 min gradient. The gradient was held at 4 % B for 3 min, followed by a linear 60 min increase to 50 % B. After 10 min at 90 % B, the column was washed at 4 % B for 15 min. A source temperature of 250 °C was applied and full-scan MS spectra were acquired at a resolution of 70,000, an AGC target of 3 · 106 and a maximum injection time (IT) of 100 ms between 300-1500 m/z. For higher-energy collision dissociation, the 10 most intense precursor ions were selected and fragmented with a normalized collision energy of 27. MS2 spectra were acquired with a resolution of 17,500, an AGC target of 1 · 105, and an IT of 50 ms over a scan range of 200-2000 m/z.

The raw MS files were processed with Proteome Discoverer (V3.0.1.27, Thermo Fisher Scientific, Waltham, MA, USA). MS/MS spectra were searched using the CHIMERYS search algorithm against the protein database of the human proteome (UniProt July 2023) supplemented by the isoforms of APOE and a data set containing common contaminants (cRAP, May 2021). The search was performed using tryptic protease specificity allowing 2 missed cleavages and peptide length between 7 to 30 amino acids. Oxidation of methionine residues (M, + 15.995 Da) was set as a variable modification and carbamidomethylation of cysteine residues (C, + 57.021 Da) as a static modification. Only proteins with at least two identified peptides and proteins that showed twofold or higher enrichment compared with the untreated or mock control were included in the final lists.

Seahorse ATP rate assay

The isoform-dependent effects on the mitochondrial and glycolytic ATP production rate was assessed in living APOE3- and APOE4-transfected Huh7 cells employing Agilent Seahorse technology including specialized consumables and reagents (Agilent, Santa Clara, CA, USA). The cells were seeded in 10 cm petri dishes for APOE transfection, collected by trypsination after 24 hours, and seeded into XFe96 microplates at a density of 30,000 cells per well. The cells were allowed to adhere for one hour at room temperature before being transferred to the incubator for further 16 hours. The next day, the cells were washed, and then incubated in Seahorse XF RPMI Medium for one hour at 37 °C without CO_2. Prior to the ATP Rate Assay, 2-deoxyglucose (2DG; Thermo Fisher Scientific, Vilnius, Lithuania) was injected at a final concentration of 50 mM to inhibit glycolytic ATP production. Control cells were injected with Seahorse XF RPMI Medium. The analysis was conducted in the Seahorse XF Pro Analyzer (Seahorse Bioscience, Agilent, Santa Clara, CA, USA) according to the manufacturer’s instructions. Immediately after the Seahorse analysis, the cell nuclei were stained with 1 μg/ml 4',6-Diamidino-2-phenylindol (DAPI; Sigma, Steinheim, Germany) in DPBS and fluorescence was measured at 360 nm in a Tecan infinite F200 microplate reader (Tecan, Grödig, Austria). DAPI fluorescence was used as a measure of cell count and for normalizing the ATP production rate.

Mitochondrial DNA analysis

Total DNA from livers of human APOE-targeted replacement mice was isolated using the DNeasy Blood & Tissue Kit (Qiagen, Hilden, Germany) following the manufacturer's instructions. DNA concentration was determined photometrically at 260 nm using the Nanodrop 2000 Spectrophotometer (Thermo Fisher Scientific, Waltham, MA, USA) and stored at −20 °C until use. The qPCR was performed using the PowerTrack SYBR Green Master Mix (Thermo Fisher Scientific, Vilnius, Lithuania) on a Rotorgene 6000 cycler (Corbett Life Science, Sydney, Australia). Primer sequences targeting the mitochondrially encoded NADH dehydrogenase 1 (*Nd1*) (5′-3′: AAGGAGAATCAGAATTAGTATCAGGGTT; 3′-5′ TAGTACTCTGCTATAAAGAATAACGCGAAT; [74]) and mitochondrial 16S ribosomal RNA (*16SrRNA*) (5′-3′: CCGCAAGGGAAAGATGAAAGAC; 3′-5′: TCGTTTGGTTTCGGGGTTTC; [75]), along the nuclear gene hexokinase 2 (*Hk2*) (5′-3′: GCCAGCCTCTCCTGATTTTAGTGT; 3′-5′: GGGAACACAAAAGACCTCTTCTGG; [75]) were extracted from the literature. purch. Primers targeting the nuclear encoded glyceraldehyde 3-phosphate dehydrogenase (*Gapdh*) (5′-3′: CCGCATCTTCTTGTGCAGT; 3′-5′: GGCAACAATCTCCACTTTGC) were newly designed using the free online Primer3web software (version 4.1.0). All primers were purchased from Eurofins MWG (Ebersberg, Germany). Target mitochondrial DNA (mtDNA) concentration was calculated using an external standard curve and normalized to nuclear DNA content.
